# Satisfaction of beneficiaries with community-based health insurance and associated factors in Legambo District, North-East Ethiopia: a cross-sectional study

**DOI:** 10.3389/fpubh.2023.1127755

**Published:** 2023-05-16

**Authors:** Melaknesh Minda Getaneh, Ewunetie Mekashaw Bayked, Birhanu Demeke Workneh, Mesfin Haile Kahissay

**Affiliations:** ^1^Department of Capacity Building and Operational Research, Ethiopian Pharmaceuticals Supply Services (EPSS), Dessie, Ethiopia; ^2^Department of Pharmacy, College of Medicine and Health Sciences (CMHS), Wollo University, Dessie, Ethiopia; ^3^Department of Pharmaceutics and Social Pharmacy, School of Pharmacy, College of Health Sciences, Addis Ababa University, Addis Ababa, Ethiopia

**Keywords:** community-based health insurance, beneficiaries, satisfaction, factors, Ethiopia

## Abstract

**Background:**

The fundamental concept of community-based health insurance is to strengthen the healthcare financing system to access universal healthcare by reducing costly risk-coping strategies. The scheme’s sustainability and the quality of services provided by it are highly dependent on the satisfaction of its beneficiaries. Despite beneficiaries’ satisfaction being the key determinant for providing evidence for policy revision and decision-making, it has often been neglected. Therefore, the study investigated the community-based health insurance beneficiaries’ satisfaction and associated factors in Legambo district, North-East Ethiopia.

**Methods:**

The study was conducted in the Legambo district with a community-based cross-sectional study design from October to November 2019. The data were collected from 838 households that had been the beneficiaries of the scheme using multi-stage and systematic random sampling. Twelve trained data collectors were employed and gathered the data using a pre-tested, structured questionnaire. We ran descriptive, bivariate, and logistic regression analyses. A value of p less than 0.05 with a 95% CI was used in multivariate logistic regression to determine the association of variables with the beneficiaries’ satisfaction.

**Results:**

The overall satisfaction level of the beneficiaries of the scheme was 58.6% and was associated with the following factors: merchandize (AOR = 1.92, 95% CI = 1.02–3.63), living in rural areas (AOR = 1.52, 95% CI = 1.02–2.27), an early office opening time (AOR = 3.81, 95% CI = 2.04–7.10), a short time interval to use benefit packages (AOR = 4.85, 95% CI = 2.08–11.31), an inexpensive membership premium (AOR =10.58, 95% CI = 3.56–31.44), availability of laboratory services (AOR =2.95, 95% CI = 1.71–5.09), presence of referral services (AOR =1.93, 95% CI = 1.33–2.80), having immediate care at health facilities (AOR = 1.73, 95% CI = 1.01–2.97) and non-compulsory enrolment (AOR = 6.31, 95% CI = 1.64–24.20).

**Conclusion:**

The beneficiaries’ satisfaction with the scheme was suboptimal and found to be determined by occupation, residence, laboratory and referral services, immediate care, office opening time, time interval to use benefit packages, premium amount, and situation of enrollment, most of which are service-related variables. Thus, to improve the satisfaction level, the stakeholders that should work hard seem to be the health insurance agency (the insurer) and the health facilities (the provider or supplier).

## 1. Introduction

Health security and improvements in health outcomes are integral parts of the global commitment to poverty reduction (1). That is why more than 800 million people (12%) in the world spend at least 10% of their household budgets to pay for healthcare (2). Consequently, universal health coverage (UHC) is a priority issue on the Sustainable Development Goals (SDGs) and global development agenda (3). A tax-funded health system may be difficult to establish in developing countries due to a lack of a strong tax base and a lack of institutional capacity to collect taxes (4). As a result, without health insurance, a large population will remain overly dependent on direct out-of-pocket (OOP) expenses (5). Globally, OOP medical spending results in massive financial barriers to accessing healthcare and impoverished lives in lower socioeconomic households. Approximately 44 million households (over 150 million people) face financial problems due to healthcare expenditures. About 25 million households are in deep poverty. Thus, the provision of affordable healthcare to the population in low- and middle-income countries is a persistent development issue (3).

Ethiopia’s healthcare system is funded by a variety of sources, including loans and gifts from all over the world (46.8%), the Ethiopian government (16.5%), out-of-pocket payments (35.7%), and others (0.9%). There are two health insurance systems in Ethiopia: social health insurance (SHI) for the formal sector and community-based health insurance (CBHI) for the informal population (6). The implementation of SHI, however, has been repeatedly delayed despite being anticipated to be completely operational in 2014 due to strong opposition from public employees, notably health professionals (7).

The CBHI is a non-profit mechanism for insuring the poor to enable them to access basic healthcare services (8). It applies the principles of insurance in the social context and is guided by the communities’ preferences and based on their structures and arrangements. It is especially useful in reaching the poor rural residents and the informal sector that are unable to pay OOP costs in developing countries like Ethiopia (9). The state of healthcare financing in Ethiopia has been characterized by low government spending and insignificant participation by the private sector (10). Ethiopia’s healthcare financing heavily depends on OOP expenditure (11). Consequently, only about 50% of the population has access to basic health services (10).

Ethiopia launched CBHI in 2011 as a risk protection mechanism for the rural and informal sectors (12). Except for false teeth, eyeglasses, and cosmetic procedures, its benefit package covers all outpatient and inpatient services at the health center and nearby hospital levels (13). For patients following a referral system, it provides them with primary, secondary, and tertiary care. In public health facilities, it offers free care that is reimbursed through a fee-for-service system (6).

Enrollment is on a household basis to reduce the possibility of adverse selection. For all members, the federal government offers a general subsidy of 25%. Using their own funds, districts and regions support a solidarity fund for the poor, who make up an estimated 10% of the population. Fee-for-service is the mechanism used to pay the provider (13). The Ethiopian government revised the CBHI plan premium from 240 Birr to 410 Birr per household per year since the prior contribution plus 25% government subsidies for all members were insufficient to meet the costs of healthcare services (14).

CBHI coverage should be designed to improve the quality of care, which impacts clients’ satisfaction, which in turn affects its sustainability (15). This is because not only could CBHI enrollment guarantee quality healthcare services (16), but beneficiaries’ satisfaction is also an important indicator of healthcare quality and is often associated with greater adherence to medical technology, health service utilization, and health outcomes (17). High satisfaction with CBHI encourages its scale-up (18).

Beneficiaries’ satisfaction is a multi-dimensional healthcare outcome affected by many variables, including patient, physician, and system-related factors (19). According to Haile et al. (20), beneficiaries’ satisfaction is influenced by family size, knowledge about the benefits packages, friendliness with healthcare providers, privacy, and confidentiality; getting prescribed drugs; availability of laboratory services; perceived cleanness of health facilities; length of waiting time; the way queries were dealt with by staff; and agreement with the benefits packages of the CBHI (20).

Patients, according to Geng et al. (21), are key stakeholders and beneficiaries of health insurance schemes. Their views are crucial for influencing health insurance policies, providing feedback on the responsiveness and quality of insurance programs, and bringing accountability and transparency to the decision-making process for insurance policies (21).

The effectiveness of the health system therefore depends on improvements in access to quality care and client satisfaction. Satisfaction surveys are important to solve the problems of access and performance. Patient satisfaction is a central issue that is entangled with strategic health services decisions. These surveys are essential instruments for helping government agencies identify target groups, clarify objectives, define measures of performance, and develop performance information systems (22). However, globally, there were limited studies regarding the factors determining the satisfaction level of CBHI beneficiaries (23), which was also true for Ethiopia. As a result, the study sought to investigate the level of satisfaction among CBHI beneficiaries and associated factors in Legambo District, North-East Ethiopia.

## 2. Materials and methods

### 2.1. Study design and setting

A cross-sectional study was used to assess the satisfaction level and associated factors of beneficiaries of CBHI. The study was conducted from October to November 2019 among households in Legambo district, South Wollo Zone, and the Amhara region. The district is 501 km from Addis Ababa, the capital city of Ethiopia. It is the second-most populous district in the South Wollo Zone. According to the 2007 population and housing census of Ethiopia, the district has a population of 165,026 (81,268 men and 83,758 women), of which only 4.4% are urban inhabitants. It has a total of 39,078 households (an average of 4.22 persons per household). Around 92.99% of the population were Muslim, and the rest were Orthodox Christians (24). It has 37 rural and three urban kebeles. The common economic activity of the population is agriculture (farming and animal breeding). The CBHI was launched in 2017 in the district. In 2019, the plan had 83% of members enrolled. Legambo was ranked second in the affiliated zone (25). This was what inspired the principal investigators to measure the level of beneficiary satisfaction and associated factors of CBHI.

### 2.2. Participants and sample

The target and study populations, respectively, were all households using CBHI in Legambo district and the sampled CBHI scheme users who live in the 12 randomly selected kebeles. All households headed by permanent residents in the district who were members of CBHI were included in the study. Household heads who were not willing to participate, not available at the time of data collection, seriously ill, or practicing as formal employees were excluded. Moreover, households that were not headed by adults (less than 18 years of age) were also excluded.

The sample size was calculated using Epi-Info version 7 and determined by the single population proportion formula. Accordingly, using 54.7% of satisfaction with the CBHI in South-West Ethiopia (26), a confidence level of 95%, and a 5% margin of error, 381 samples were obtained as follows:


$$n=\frac{z^{2}\;\left(p\right)\left(q\right)}{d^{2}}$$

where the given variables, n, z, p, q, and d, are the sample size, standard deviation, satisfaction level, non-satisfaction level, and margin of error, respectively. Then,


$$n=\frac{{\left(1.96\right)}^{2}\;\left(0.547\right)\left(0.453\right)}{{\left(0.05\right)}^{2}}=381$$

Since the sampling procedure involves two stages, to account for the design effect, two times the calculated sample size (381 × 2 = 762) was taken, as shown in the following formula:


$$n=2\frac{{\left(1.96\right)}^{2}\;\left(0.547\right)\left(0.453\right)}{{\left(0.05\right)}^{2}}=762$$

Finally, by adding a 10% non-response rate (10% × 762), the total sample size was determined to be:


$$n=\left(762\right)+\left(0.1\times 762\right)=838$$

The participants were selected using a two-stage sampling technique. First, 12 out of 40 rural kebeles were selected using the lottery method. Then, the sample size was proportionally allocated among the selected kebeles based on the number of households. Finally, a systematic sampling technique was used to access samples based on the order of registration of the heads of each household in the health extension workers’ (HEWs’) records at the health post of each kebele.

### 2.3. Data collection procedures

A structured, interviewer-administered questionnaire, extracted from various pieces of literature, was used for data collection. It had two main parts: socio-demography and determinants of CBHI satisfaction (knowledge of the CBHI scheme, the CBHI process, and management-related factors; factors related to health services provision; experiences related to CBHI members; factors related to benefit packages). It was initially prepared in English, translated into Amharic, and then back to English for consistency. It was pre-tested on 5% of participants (*n* = 42) in a comparable setting (Legahida district). The survey was conducted over 2 months (October to November 2019). The team consisted of four principal investigators (MMG and EMB) and two supervisors (BDW, EMB, BDW, and MHK). Twelve data collectors with a BSc in nursing and extensive data collection experience took part. They have been provided with theoretical and field training by the investigators for a half day. They used a house-to-house survey. Alternative visits were arranged for household heads who were not available at the first visit. So, when the participant was not available after two visits or was unwilling to participate, the immediate next household in the sampling frame was considered.

### 2.4. Data processing and analysis

The data were entered into Epi-Info 7 and analyzed using SPSS version 23.0. The characteristics of the participants were described using frequency, percentage, mean, mode, median, range, and standard deviation. Seven variables were used to measure respondents’ knowledge of the CBHI benefit package. If respondents answered more than four questions, they were labeled as having adequate knowledge of CBHI benefit packages. The level of households’ satisfaction with the CBHI scheme was assessed using the Likert scale. One point was given for favorable responses and zero for unfavorable responses, and the mean score was calculated. Then, the households were categorized as “not satisfied” if the score was less than the mean score or “satisfied” if it was greater than or equal to the mean. Cronbach’s alpha coefficient was calculated for the outcome variable (satisfaction with CBHI) to test the reliability of the questionnaire. The bivariate logistic regression model was used to find the association of each independent variable with the outcome variable. A value of p of less than or equal to 0.25 with a 95% confidence interval in the bivariate analysis was entered into the multivariate model. In multivariate analysis, variables with a value of p of less than 0.05 and a 95% CI were considered significantly associated with satisfaction. Hosmer and Lemeshow’s goodness-of-fit test, the Wilks test (Cronbach’s alpha), and the Wald statistic test were used to check internal consistency and model fitness.

### 2.5. Data quality control

More than two investigators participated (MMG, EMB, BDW, and MHK). The data collection tools were prepared from multiple sources. The tools were pretested on 5% of the samples in a comparable setting (the Legehida district) for their content and any ambiguity and were modified and validated accordingly. All the tools were repeatedly checked for their completeness on a daily basis.

## 3. Results

### 3.1. Socio-demographic description

In this study, 838 households were sampled. Eight hundred seven (96.3%) of the households responded, out of which 716 (88.7%) respondents utilized one or more health services. The majority, 599 (74.2%), were male. Most of the participants (253, or 31.4%) were in the age category of 40–49 years. The mean age of the participants (age ± SD) was 42.6 ± 11.7 with a range of 62 years. Two-fifths (39.4%) of them had no education. The average annual income was $675 (Table 1).

**Table 1:** Sociodemographic characteristics of participants in Legambo district, North-East Ethiopia (*n* = 807), 2019.

Variables	Category	Frequency (*N*)	Percent (%)
Sex	Male	599	74.2
	Female	208	25.8
Age	<30	145	18
	30–39	196	24.3
	40–49	253	31.4
	≥50	213	26.4
Marital status	Married	712	88.2
	Single	25	3.1
	Divorced	48	5.9
	Widowed	22	2.7
Family size	1–5	617	76.5
	>5	190	23.5
Religion	Protestant	14	1.7
	Orthodox	94	11.6
	Muslim	694	86.0
	Catholic	5	0.6
Educational level	Not able to read and write	318	39.4
	Able to read and write	302	37.4
	Primary education	133	16.5
	Secondary education	48	5.9
	College education and above	6	0.7
Major occupation	Farmer	699	86.6
	Merchant	79	9.8
	Unemployed	27	3.3
	Handcraft	2	0.2
Residence	Urban	195	24.2
	Rural	612	75.8
Estimated annual income	<$250	106	13.1
	$250–$500	245	30.4
	>$500	456	56.5

### 3.2. Households’ knowledge of CBHI benefit packages in the CBHI scheme

Most of the participants had appreciable awareness regarding the scheme; 93.1% of the respondents were found to have adequate knowledge (Table 2).

**Table 2:** Knowledge of CBHI benefit packages among households in Legambo district, North East Ethiopia (*n* = 807), 2019.

Variables	Category	Frequency	Percent %
CBHI is a good way of helping clients with health expenditure	Yes	775	96.0
	No	32	4.0
CBHI covers only care from public health institutions	Yes	662	82.0
	No	145	18.0
CBHI covers transportation fee	Yes	82	10.4
	No	723	89.6
CBHI covers only care within the country	Yes	699	86.6
	No	108	13.4
CBHI covers outpatient care	Yes	769	95.3
	No	38	4.7
CBHI covers inpatient care	Yes	767	95.0
	No	40	5.0
CBHI covers medical care for cosmetic values	Yes	44	5.5
	No	763	94.5
Adequate knowledge of CBHI benefit packages	Yes	751	93.1
	No	56	6.9

### 3.3. Health service-related variables

**Figure 1:**
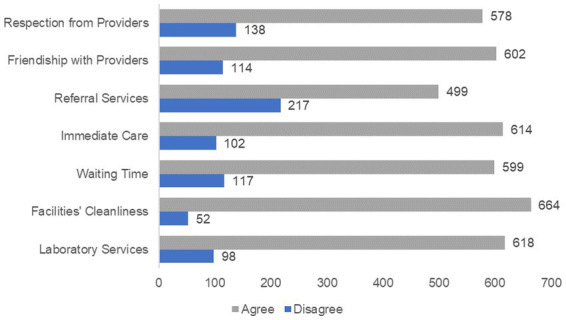
Health service-related variables for CBHI beneficiaries’ satisfaction in Legambo district, North-East Ethiopia (*n* = 716), 2019.

Out of 716 respondents, most agreed with the availability of laboratory services (86.3%), appropriate facility cleanliness (92.7%), immediate care (85.8%), and referral services (69.8%). Short waiting time (83.7%), healthcare providers’ friendship or relationship (84.1%), and respecting (80.7%) were also agreed upon by the majority of participants (Figure 1).

### 3.4. CBHI process and management-related variables

**Figure 2:**
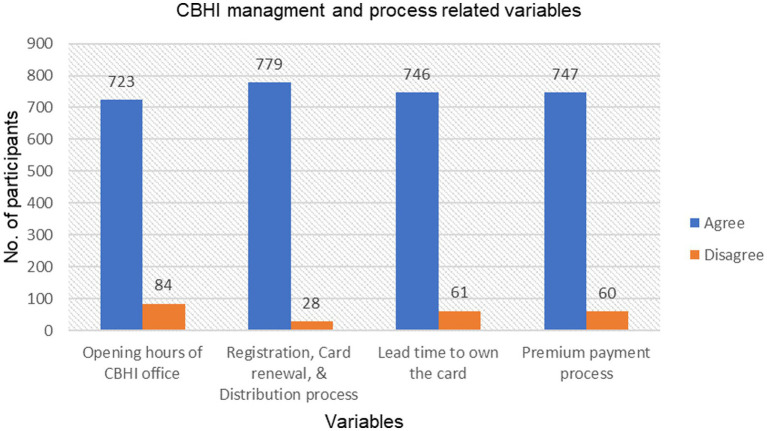
The CBHI process and management-related characteristics of respondents enrolled in the scheme in Legambo district, North-East Ethiopia (*n* = 807), 2019.

Of the 807 respondents, most agreed on the opening hours of the CBHI office (89.6%), membership registration, card renewal, and distribution processes, i.e., the time gap to get the card after registration or re-registration (96.5%), and paying the premium (92.6%). Some (7.3%) were neutral about membership registration, card renewal, and distribution processes (Figure 2).

### 3.5. Experience of household heads in CBHI scheme

Of all participants, most (97.0%) enrolled in the scheme voluntarily. Both hospitals and health centers were visited to get healthcare services. However, health centers were the most frequently visited (79.9%). Most (90.7%) were happy with the mandated health facilities that provide CBHI benefit packages. The majority (88.7%) of participants reported that at least one of their family members had fallen sick and visited a health institution within the last 12 months. The majority of household members (94.9%) enrolled before a year. Of those who visited health facilities, 71.5% have ever been prescribed drugs. More than three-fifths (63.3%) of respondents have participated in CBHI-related meetings, and 41.5% have ever discussed CBHI with the scheme’s managers. The respondents who preferred to pay the premium one time, two times, and three times per year were 11.6, 31.1, and 57.2%, respectively, but all respondents have been paying annually (Table 3).

**Table 3:** Households’ experiences with the CBHI scheme in Legambo district, North-East Ethiopia (*n* = 807), 2019.

Variables	Category	Frequency	Percent %
Voluntary enrolment in the CBHI scheme	Yes	783	97.0
	No	24	3.0
Health institution/facility visited	Only hospital	43	5.3
	Hospital and health center	28	3.5
	Only health center	645	79.9
Frequency of health facility visiting	Once	146	18.1
	Twice	167	20.7
	3 Times	164	20.3
	>3 Times	239	29.6
Length of enrolment	*<*12 Months	41	5.1
	≥12 Months	766	94.9
Happy with the permitted health institutions	Yes	732	90.7
	No	75	9.3
Got prescribed drugs	Yes	557	71.5
	No	222	28.5
Participation in CBHI-related meeting	Yes	511	63.3
	No	296	36.7
Discussion with CBHI managers	Yes	335	41.5
	No	472	58.5
How many times do you prefer to pay the premium for CBHI	Once	94	11.6
	Twice	251	31.1
	3 Times	462	57.2

### 3.6. Level of satisfaction with the CBHI scheme

**Figure 3:**
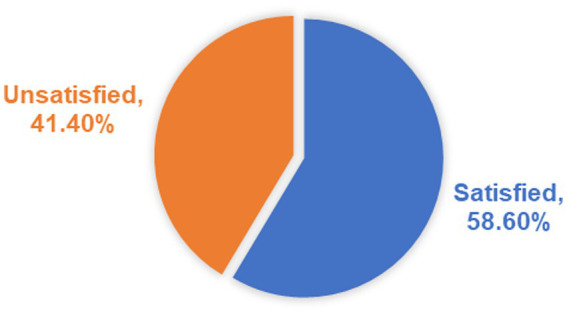
Households’ level of satisfaction with the CBHI scheme in Legambo district, North-East Ethiopia (*n* = 807), 2019.

The level of household satisfaction with the CBHI scheme was rated using five questions, each having five points on a Likert scale. Respondents had a minimum of 5 and a maximum of 25 points on the CBHI scheme satisfaction score. The mean satisfaction score was 3.977. Then, households were categorized as satisfied if the score was above the mean and not satisfied if the score was below the mean. Consequently, 473 of the 807 total respondents were satisfied, providing an overall satisfaction of 58.6% (Figure 3).

### 3.7. Determinants of CBHI scheme beneficiaries’ satisfaction

Without controlling confounding, some variables were significantly associated with the members’ satisfaction with the CBHI scheme. At the bivariate level of analysis, lead time to use the benefit package, CBHI offices’ opening times, membership registration, renewal, and card distribution, premium, members’ awareness, availability of laboratory services, CBHI members’ beliefs toward healthcare providers’ respect and friendliness, facility cleanliness, waiting time, immediate care, referral service, having prescription drugs, and type of facility were significantly associated with households’ satisfaction with the CBHI scheme (*p* < 0.05) (Table 4).

**Table 4:** Factors affecting beneficiaries’ satisfaction with the CBHI Scheme in Legambo district, North-East Ethiopia (*n* = 807), 2019.

Variables	Overall satisfaction		OR (95% CI)		*p*-value
	Satisfied, *N* (%)	Not satisfied, N (%)	COR (95% CI)	AOR (95% CI)	
*Household head’s occupation*					
Farmer	382 (54.6)	317 (53.6)	1	1	
Merchant	44 (55.7)	35 (44.3)	0.959 (0.6–1.531)	1.92 (1.02–3.63)	0.044*
Unemployed	11 (40.7)	16 (59.3)	1.753 (0.802–3.831)	0.38 (0.15–0.97)	0.044*
Other (specify)	1 (50)	1 (50)	1.205 (0.075–19.343)		
*CBHI offices opening time*					
Disagree	16 (19.0)	68 (80.1)	1	1	
Agree	457 (63.2)	266 (36.8)	7.30 (4.15–12.85)	3.81 (2.04–7.10)	0.00*
*Time interval to use benefit packages*					
Disagree	8 (13.1)	53 (86.8)	1	1	
Agree	465 (62.3)	281 (37.7)	10.96 (5.14–23.85)	4.85 (2.08–11.31)	0.00*
*Premium payment*					
Disagree	4 (6.7)	56 (93.3)	1	1	
Agree	469 (62.8)	278 (37.2)	23.6 (8.47–65.5)	10.58 (3.56–31.44)	0.00*
*Laboratory service*					
Disagree	25 (25.5)	73 (74.5)	1	1	
Agree	385 (62.3)	233 (37.7)	4.83 (2.98–7.82)	2.95 (1.71–5.09)	0.00*
*Referral service*					
Disagree	89 (41.0)	128 (59)	1	1	
Agree	321 (64.3)	178 (35.7)	2.59 (1.87–3.6)	1.93 (1.33–2.80)	0.001*
*Immediate care*					
Disagree	29 (28.4)	73 (71.6)	1	1	
Agree	381 (62.1)	233 (37.9)	4.12 (2.6–6.52)	1.73 (1.01–2.97)	0.048*
*Respection by providers*					
Disagree	53 (38.4)	85 (61.6)	1	1	
Agree	357 (61.8)	221 (38.2)	2.59 (1.77–3.40)	0.65 (0.39–1.18)	0.13
*Provider friendliness*					
Disagree	38 (33.3)	76 (66.7)	1	1	
Agree	372 (61.8)	230 (38.2)	3.24 (2.12–4.94)	0.96 (0.53–1.74)	0.89
*Facility cleanliness*					
Disagree	15 (28.8)	37 (71.2)	1	1	
Agree	395 (59.5)	269 (40.5)	3.622 (1.949–6.731)	1.47 (0.69–3.12)	0.32
*Waiting time*					
Disagree	42 (35.9)	75 (64.1)	1	1	
Agree	368 (83.1)	75 (16.9)	2.845 (1.884–4.295)	0.91 (0.51–1.64)	0.76
*Residence*					
Urban	104 (53.3)	91 (46.7)	1	1	
Rural	369 (60.3)	243 (39.7)	1.34 (0.96–1.84)	1.52 (1.02–2.27)	0.039*
*Enrollment status (Voluntary)*					
No	3 (12.5)	21 (87.5)	1	1	
Yes	470 (60)	313 (40)	10.51 (3.11–35.54)	6.31 (1.64–24.20)	0.007*
*Prescription drugs*					
No	94 (42.3)	128 (57.7)	1	1	
Yes	359 (64.5)	198 (35.5)	2.469 (1.797–3.392)	1.35 (0.89–2.05)	0.16
*Knowledge*					
No	25 (44.6)	31 (55.4)	1	1	
Yes	448 (59.7)	303 (40.3)	1.83 (1.06–3.17)	1.09 (0.51–2.33)	0.82

1 Reference category, *Significant at *p* < 0.05.

In the multivariate analysis, residence (*p* = 0.039), occupation (*p* = 0.044), premium (*p* = 0.001), availability of laboratory services (*p* = 0.001), CBHI office opening time (*p* = 0.001), lead time to get a benefit package (*p* = 0.001), voluntary enrolment for CBHI (*p* = 0.007), amount of premium (*p* = 0.001), and referral service (*p* = 0.001) were significantly associated with the scheme’s satisfaction.

Merchant respondents were 1.92 times more likely to be satisfied than farmers (AOR = 1.92, 95% CI = 1.02–3.63), but unemployed respondents were 0.38 times less likely to be satisfied than farmers (AOR = 0.38, 95% CI = 0.15–0.97). The beneficiaries that appreciated the scheme’s official opening time were 3.81 times more likely to be satisfied than those who did not (AOR = 3.81, 95% CI = 2.04–7.10). The respondents who were comfortable with the lead time to get the benefits package were 4.85 times more likely to be satisfied than those who were uncomfortable (AOR = 4.85, 95% CI = 2.08–11.31).

Rural respondents were 1.52 times more likely to be satisfied than urban respondents (AOR = 1.52, 95% CI = 1.02–2.27). The users who were enrolled voluntarily were 6.31 times more likely to be satisfied than those who were forced to enroll (AOR = 6.31, 95% CI = 1.64–24.20).

Respondents who agreed with paying a premium were 10.6 times more likely to be satisfied than respondents who did not agree (AOR = 10.58, 95% CI = 3.56–31.44). Those who received laboratory services were 2.95 times more likely to be satisfied as compared to those who did not (AOR = 2.95, 95% CI = 1.71–5.09). Those who had referral services were 1.93 times more likely to be satisfied than those who did not (AOR = 1.93, 95% CI = 1.33–2.80). Respondents who had gotten immediate care at health facilities were 1.73 times more likely to be satisfied than those who did not (AOR = 1.73, 95% CI = 1.01–2.97).

## 4. Discussion

The WHO has been advocating UHC to curtail OOP payments, improve access to health services, and reduce financial catastrophes (27). To do so, many developing countries have introduced CBHI since the 1990s to strengthen healthcare financing and improve access to healthcare by reducing costly risk-coping strategies (28). However, its uptake has been challenged by a variety of factors, including satisfaction (29), demographic, socio-economic, health status, and health services issues (30). Moreover, treatment outcome and patient satisfaction are inseparable (31).

Despite the fact that beneficiaries’ satisfaction and the factors associated with it have been known to provide evidence for policy revision and decision-making, CBHI beneficiaries’ satisfaction with the scheme has often been neglected (22), which this study sought to investigate and discovered that the satisfaction level of CBHI beneficiaries was 58.60%, which is slightly comparable, higher, and lower than studies in South-West Ethiopia (54.7%) (26), Nigeria (42.1%) (22), and southern Ethiopia (91.38%) (32), respectively.

The study revealed that the satisfaction level has been influenced by socio-demographic, health service provision, and CBHI process and management-related variables. Regarding the socio-demographic variables, occupation and residence were significant predictors of satisfaction with the CBHI, as also reported by another study (33). However, other variables such as age, education, marital status, sex, and income were not significant predictors, which is consistent with studies in Ethiopia (26), Nigeria (34), and India (15). Nonetheless, income (35), age (33, 35), marital status (22, 33), gender, education, self-perceived health status, and type of household’s plan (33) were also found to be significant factors. Similar to a finding in Turkey (33) but opposite to a report in Nigeria (34), this study found that the occupation of the scheme’s members had a significant association with their satisfaction. Accordingly, farmers were less satisfied than merchants but more satisfied than unemployed beneficiaries. This might be because as income increases, the affordability of the premium becomes more likely. However, currently, all regional CBHI schemes charge similar premiums and registration fees regardless of household income and location, which will not be fair, compromise the financial sustainability of the scheme (12), and result in the exclusion of the poorest of the poor (36), who may be motivated to prepay if their contributions are supplemented by government or donor agencies (29), or if they are registered as indigents (12). So, to ensure equitability, the premiums should be based on income levels (37). On the other hand, as livestock size increased with income, the farmers’ interest in implementing the scheme decreased because the livestock were considered to be reserved assets (30). Despite the far distance, this study showed that rural respondents were more satisfied than urban dwellers, which did not deter households from joining the scheme (12); i.e., the rural and informal sectors, in particular, benefited from this program (32). In contrast, in urban areas, the risk of moral hazards was found to be more likely (12), which might be a reason for poor equity of care and dissatisfaction.

Regarding health service provision, the overall satisfaction of the respondents was 57.26%, and having laboratory, referral, and immediate care services was significantly associated with satisfaction. This satisfaction level was a little higher than the studies in Ethiopia (54.1%) (35) and Turkey (55.9%) (33), but lower than most of the studies in Ethiopia: 63.4% (38), 79.4% (39), and 80% (40), and in Nigeria: 73.1% (41), 80.6% (42) and (75.5%) (31). The beneficiaries’ satisfaction could be dependent on various domains: duration, process, availability, access, continuity, and quality of services; and the attitude of health personnel (humaneness) (33, 42); availability of doctors and medicines; and the patient’s recovery being the main reasons (15). However, the delivery of health services provided by the CBHI scheme was not satisfactory in terms of quality of care, referral systems, human resources, and building facilities (43). There were also pieces of evidence that consultation and diagnosis services are much more commonly performed among non-insured patients than insured patients (39).

Beneficiaries who agreed and/or received laboratory services were more satisfied compared to those who disagreed, which is consistent with previous studies (26, 32, 35). But the mere presence of the service might not be the only case to be concerned with; rather, the availability of laboratory personnel, explanations about diagnostic tests during sample collection, cleanliness, and comfort of the latrine and waiting area should be considered as the main factors of CBHI beneficiaries’ satisfaction with medical laboratory services (44).

Referral service was another factor affecting the beneficiaries’ satisfaction with the CBHI scheme; i.e., the beneficiaries who got referral service were more satisfied than those who did not. This might be because many people expect the quality of care to be superior at hospitals. So, they prefer to go directly to the secondary level if no referral is required, i.e., they demand a referral before using the services of a lower-level facility. As the delivery of care is more expensive at the hospitals, CBHI can worsen existing inefficiencies in the absence of a mandatory referral system (12, 45, 46). So, since access to the hospital has required a referral from the first-contact primary care provider (gatekeeper), self-referral costs could not be covered by the scheme (12, 46). Yet, beneficiaries are allowed to access hospitals without penalty with a health center referral, but members who bypass the referral system are required to pay an OOP bypass fee of 50% (13). This is also planned to be omitted, as the CBHI scheme will not cover the cost of health services for any beneficiary who uses the health service without following the referral system (47). This might result in great dissatisfaction unless both providers and CBHI members have become assertive in demanding immediate referral (12).

Contrary to a study that reported waiting time as an unnecessary parameter for satisfaction (48), beneficiaries who perceived, expected, or received immediate care were more satisfied than those who did not. Whereas extended time to get healthcare service neither leads to satisfaction nor adherence to the CBHI scheme (49), beneficiaries spend prolonged time in the medical records, accounting, and pharmacy sections (31). The worst situation is that insured patients have been waiting longer at health facilities than uninsured patients and are being discriminated against by providers (50). This might result in extreme dissatisfaction with the scheme and its collapse unless the district health offices work better, particularly around the waiting time for patient-provider interaction (38).

In line with the report of another study (32), the other important finding of this study was that there was a significant association between the CBHI process and management factors and CBHI satisfaction, particularly CBHI office opening time, time interval to use the benefit package (length of time between registration and service use or waiting period), and amount of payment. Ease of registration and payment, quality of service, and short waiting times at the insurance administrative office are positive predictors of satisfaction with the CBHI scheme (42). While satisfaction with CBHI for the cost of care is defined by participants reporting satisfaction with the premium paid, the share of costs, and high medical bill protection (51), the premium load was decided only by members’ family size without considering their level of income (12), which might lead to dissatisfaction with the accessibility of premium prices (52). But, in reality, since CBHI schemes consist of poor households, their ability to raise significant resources to pay for healthcare is limited by the community’s overall income, their exposure to OOP payments when not enrolled, and the availability and size of subsidies (29). As a result, if these factors are overlooked, unaffordable premiums and inconvenient models of premium payment could remain the main reasons for low adherence to the CBHI scheme (49). To do so, the federal government has provided a 25% general subsidy for all members (13). After paying the premium, time intervals to use benefit packages (the waiting period) and CBHI office opening times were the main factors in determining CBHI members’ satisfaction with the scheme (32). Members should wait 1 month before they can use covered services (all outpatient and inpatient services except false teeth, eyeglasses, and cosmetic procedures) (13).

The study found that beneficiaries who were enrolled voluntarily were more satisfied than those who were enforced, which was not a significant factor in a similar study (32). Direct community involvement in the design and management of the scheme has increased the satisfaction level (29); i.e., when the scheme administrators tend to be responsive to the community’s preferences, the overall satisfaction with the scheme’s services increases (53).

Members’ knowledge about the benefit packages of the scheme was high (93.1%), in line with a study (54), but in contrast to other studies (22, 26, 35), there was no significant association with satisfaction; yet, greater understanding and experiences with it are associated with lower dropout rates (55). This is higher than the report from an earlier pilot study (45.7%) (26). The difference might be because the concept of CBHI was new when this pilot study was conducted. However, the 2015 final report of the evaluation of CBHI pilot schemes by the Ethiopian Health Insurance Services (EHIS) showed that knowledge about the scheme was 95% for both members and non-members, which was the highest and attained through the dissemination of information through informed neighbors, CBHI officials, or house-to-house sensitization (12); that also seemed to be an effective means to improve the beneficiaries’ satisfaction with the scheme.

### 4.1. Policy and practical implications

A country’s economy relies on its overall citizens’ health, which is measured by equitable and efficient healthcare (45), emphasizing strong primary healthcare (PHC) in achieving UHC (56–58), one of the most prominent global health policies (56). PHC avoids costlier future care with better outcomes and higher patient satisfaction (59) by narrowing the gap between socially deprived and advantaged populations. PHC, with financial protection mechanisms, is the gold pathway to achieve UHC (56). To do so, healthcare financing (HCF), particularly CBHI, is considered a sustainable mechanism to create equitable access (60).

Service quality is a necessary precondition for successful implementation of CBHI (45) and is measured by the process (client-provider interaction) and outcome (client satisfaction) factors (61, 62); the widely used metric is the latter (63). Decisions by the healthcare provider and their attitude have a great impact on the demand for CBHI and its financial balance (45). That is why satisfaction with CBHI is very high, with more cohesive provider-patient relationships (64). This implies that provider behavior is a major determinant of beneficiaries’ satisfaction. Thus, provider selection (public vs. private) by policymakers (62), particularly for the EHIS, is an important design issue.

Besides the design, management capacity is imperative to run the scheme on a routine basis and make necessary revisions (45). Initially, health insurance schemes paid little attention to consumer satisfaction or even what consumers desired (46). In any case, since consumer satisfaction and people’s preferences and perceptions are crucial determinants for the successful implementation of CBHI, initiators and managers of the scheme are expected to pay more attention to these factors (45). However, the strategic plan of EHIS excludes beneficiaries’ satisfaction, which is hereafter strongly recommended to be included (65).

### 4.2. Limitations

The study did not investigate the perceptions and experiences of stakeholders from the supply side (healthcare providers) and the CBHI agency.

### 4.3. Conclusion

The overall satisfaction level of the CBHI beneficiaries was suboptimal. Occupation, residence, laboratory and referral services, immediate care, CBHI office opening time, time interval to use benefit packages, premium amount, and condition of enrollment were found to be the significant factors affecting the members’ satisfaction with the scheme. Most of these factors are related to the insurer and health service providers, which should be taken as evidence to revise strategies or improve service by the insurer and affiliated health facilities.

## Data availability statement

The original contributions presented in the study are included in the article/supplementary material, further inquiries can be directed to the corresponding author.

## Ethics statement

The studies involving human participants were reviewed and approved by Research, Community Service, and Graduate Coordinating Office” of the College of Medicine and Health Sciences, Wollo University. The patients/participants provided their written informed consent to participate in this study.

## Author contributions

MG and EB conceived and designed the study, performed the study, analyzed and interpreted the data, contributed materials, analysis tools, or data, and wrote the paper. MK and BW conceived and designed the study, performed the study, and analyzed and interpreted the data. All authors contributed to the article and approved the submitted version.

## Conflict of interest

The authors declare that the research was conducted in the absence of any commercial or financial relationships that could be construed as a potential conflict of interest.

## Publisher’s note

All claims expressed in this article are solely those of the authors and do not necessarily represent those of their affiliated organizations, or those of the publisher, the editors and the reviewers. Any product that may be evaluated in this article, or claim that may be made by its manufacturer, is not guaranteed or endorsed by the publisher.
